# Rapid Stress System Drives Chemical Transfer of Fear from Sender to Receiver

**DOI:** 10.1371/journal.pone.0118211

**Published:** 2015-02-27

**Authors:** Jasper H. B. de Groot, Monique A. M. Smeets, Gün R. Semin

**Affiliations:** 1 Department of Social and Organizational Psychology, Faculty of Social and Behavioral Sciences, Utrecht University, Utrecht, the Netherlands; 2 Department of Psychology, Koç University, Istanbul, Turkey; 3 Instituto Superior de Psicologia Aplicada (ISPA), Instituto Universitário, Lisbon, Portugal; University of Graz, AUSTRIA

## Abstract

Humans can register another person’s fear not only with their eyes and ears, but also with their nose. Previous research has demonstrated that exposure to body odors from fearful individuals elicited implicit fear in others. The odor of fearful individuals appears to have a distinctive signature that can be produced relatively rapidly, driven by a physiological mechanism that has remained unexplored in earlier research. The apocrine sweat glands in the armpit that are responsible for chemosignal production contain receptors for adrenalin. We therefore expected that the release of adrenalin through activation of the *rapid* stress response system (i.e., the sympathetic-adrenal medullary system) is what drives the release of *fear* sweat, as opposed to activation of the slower stress response system (i.e., hypothalamus-pituitary-adrenal axis). To test this assumption, sweat was sampled while eight participants prepared for a speech. Participants had higher heart rates and produced more armpit sweat in the fast stress condition, compared to baseline and the slow stress condition. Importantly, exposure to sweat from participants in the fast stress condition induced in receivers (*N* = 31) a simulacrum of the state of the sender, evidenced by the emergence of a fearful facial expression (facial electromyography) and vigilant behavior (i.e., faster classification of emotional facial expressions).

## Introduction

Accumulating evidence has indicated that humans are capable of communicating fear via the sense of smell. Neural and behavioral data showed that exposure to body odor from fearful “senders” elicited in “receivers” a state that resembled the fearful state of the sender (i.e., *simulacrum*; e.g. [1–4]). Recently, a dynamic social communication framework was applied to chemosignaling research [3, 5], as social communication was regarded as one of three functions of human olfaction [6]. However, what previous research has not examined was the time course and physiological mechanism responsible for the release of fear chemosignals. Because apocrine sweat glands—related to chemosignal excretion [7]—are activated by adrenalin [8], we examined whether the release of so-called fear chemosignals is driven by the activation of a rapid physiological concomitant of fear, the adrenalin-releasing sympathetic-adrenal medullary (SAM) stress system. Since an empirical test of this assumption had not been forthcoming, the current research was tailored to answer the question: Is there a rapid physiological process that drives the release of a distinctive fear odor signature leading a receiver to display a simulacrum of the state of the sender?

Even though most humans nowadays live in a protected and considerably safe environment, modern societal challenges can still trigger the rather primitive response to fight or flee [9]. By serving to prepare an individual for action toward or away from perceived threat, the fight/flight response is an essential mechanism in the survival process of the *individual*, yet particular examples (e.g., a cry for help in a dark alley) illustrate the importance of *communicating* fear-related information to other members of the species to promote survival. From an evolutionary perspective, it can be argued that survival chances of the species were increased by using multiple (i.e., visual, acoustic, and olfactory) modalities to signal danger. Compared to olfactory signals, however, auditory and visual signals have become more important over human evolutionary history. Although auditory and visual signals can be produced *intentionally* and are quickly transferred to a receiver with the speed of sound and light, the value of rapid production and secretion of chemical markers of fear would come to the forefront when the audiovisual modalities fall short (e.g., dark environments, larger distance communication, and communication while no longer being present [10]). At present, evidence suggests that body odors produced under particular circumstances may still be sufficient to induce—particularly in female receivers [11]—a simulacrum of the state of the sender [3, 12]. Although fear-related odors were demonstrated numerous times to establish synchrony between sender and receiver, what has remained unknown is the (rapid) physiological mechanism that drives the production of fear chemosignals.

An answer to this question may follow from examining the physiology of the stress response. When an event is interpreted as threatening by an individual, then it elicits not only behavioral responses such as a fearful facial expression, but also physiological responses, such as in an almost instantaneous activation of the sympathetic nervous system (SNS) [13]. As a part of the sympathetic-adrenal medullary (SAM) system, the SNS activates the adrenal medulla, resulting in the release of adrenalin. Adrenalin exerts its short-living effects on peripheral parts of the body [14], for instance by activating the apocrine sweat glands in the armpit that contain (β_2_ and β_3_) adrenoceptors [8]. The apocrine sweat glands differ from the eccrine sweat glands. Whereas the eccrine glands are used primarily for evaporative cooling, the apocrine glands are thought to be involved in chemosignaling [7]. In sum, the release of what has been labeled fear sweat is expected to be driven by relatively rapid physiological changes that accompany a system associated with the fight/flight response, namely the SAM system.

The SAM system operates in concert with another physiological stress response system, the hypothalamus-pituitary-adrenal (HPA) axis. Both physiological stress response systems co-ordinate action to deal with threatening situations in a complex manner [15]. However, what is clear is that activation of the SAM system is much more rapid (i.e., within minutes) and has short-lived effects (e.g., adrenalin circulation half-life: ~10–100 seconds) compared to HPA axis activation, which takes longer (i.e., peaks occur around 10–30 minutes after stress cessation) and effects may last for several hours [16]. Activity of the HPA axis—usually only observed under extreme circumstances—may be determined by a person’s appraisal of the threatening situation and prior experience [17]. There is no evidence to date that the main product of the HPA axis (i.e., cortisol) directly influences the most likely candidates for fear chemosignal release: the apocrine sweat glands [18]. Hence, the relatively more rapid SAM system is presumed to activate the apocrine glands through the release of adrenalin, which drives the release of a distinctive affective signature that can be picked up by and modulate the behavior of another individual.

The odor emitted by humans as a function of fear, or rather, as a reaction to a threatening stimulus, may thus have a distinctive signature. Recent evidence provided strong biochemical support for the hypothesis that the odor produced by people who were sick was qualitatively different from a placebo condition, ostensibly refuting the argument that a person’s reaction to sweat produced under particular conditions is simply based on the presence of “more of the same components” [19]. Similar to what has been demonstrated for the scent of disease [19], a qualitatively different odor may be produced rather quickly in the case of fear, facilitated by apocrine sweat that arrives on the skin along with odorless precursor molecules. Enzymes of axillary (armpit) skin bacteria transform these precursor molecules into volatile odoriferous substances [20] that can be sampled with a sniff. Hence, a quick and distinctive chemosensory cue may be released into sweat in the case of threat, the effect of which can be observed in a receiver who is expected to show a simulacrum of the sender’s experience.

Even though fear was not experimentally induced and the establishment of a simulacrum of the experience of a sender in a receiver was not examined, one study reported evidence suggestive of qualitative differences in apocrine sweat related to stress [21]. More specifically, intradermal injection of adrenalin stimulated the apocrine glands in the armpit to produce a rapid flux of apocrine secretion onto the skin surface [21] (for the procedure, see [22]). This apocrine secretion, rated as stronger and as more pungent than regular armpit sweat, was more difficult to mask with other odorants [21]. The apparent resistance to masking of stress-related apocrine sweat may illustrate the importance of the stress-related signal in transmitting information to conspecifics. However, conclusions regarding the communicative value of the signal cannot be drawn without testing whether receivers display a simulacrum of the state of the sender following exposure to sweat released as a result of experimentally induced fear.

Previous research has provided relatively consistent neural and behavioral evidence with regard to receivers exposed to sweat that was obtained from participants induced to be in a state of fear or anxious apprehension. Fear was induced by means of horror movie clips [2, 3, 11, 12, 23–25], participation in a high rope course [26–28], and tandem skydiving [1, 29, 30]. Others sampled sweat from individuals prior to an academic examination [4, 31–35] and during the administration of the Trier Social Stress Task [36, 37]. Although procedures to induce fear or anxiety differed markedly, the relatively consistent findings in receivers, ranging from amygdala activity [1] to the emergence of a fearful facial expression [3, 11, 12] and vigilant behavior [3] point to a common physiological mechanism that drives the quick release of chemosignals related to fear and anxiety.

Previous research has already shown that different stress manipulations led to increased cortisol levels [1, 33] (but see [38], for non-significant differences), heart rate (in virtually all studies, e.g. [2, 35], but see [24], for non-significant differences) and skin conductance levels (e.g. [25]) compared to the neutral condition. However, the underlying physiological mechanism had not been systematically manipulated and median sweat sampling times constituted 30 minutes. As a consequence, no evidence was documented that could substantiate the claim that the production of what has been labeled fear or anxiety sweat could be produced relatively rapidly as a function of SAM activity. If indeed the SAM system would be responsible for driving the release of fear sweat, sweat sampling procedures could be reduced by many minutes, and sampling procedures could become more effective by using measures of SAM activity. Essentially, to determine whether the release of fear/anxiety sweat by so-called senders is related to SAM activity following a threatening event rather than HPA activity, indicators of fast and slow stress need to be examined in combination.

### Present research

The present research examined whether the activation of the fight/flight response (i.e., SAM activity) following the introduction of a social stressor would lead to the production of a qualitatively different body odor that would induce a *simulacrum* of the sender’s state in a receiver. To this end, we first introduced senders to a well-validated social stressor, the Trier Social Stress Task (TSST [39]). Previous research successfully used the TSST to elicit “stress sweat” [40–42]. However, what has remained unclear has been the time frame of stress sweat production and whether receivers would show a simulacrum of the state of the sender. To test the hypothesis that SAM activity (vs. HPA axis activity) drives the chemical transfer of fear from sender to receiver, sweat sampling was divided into a “fast stress” and “slow stress” interval. First, sweat was sampled during a relaxing baseline (10 min). Next, sweat was sampled during the “fast stress” condition; participants prepared for a speech in front of an expert audience (10 min) which would result in a quick SAM response. Finally, “slow stress” sweat was sampled at a later time interval (10 min) during which SAM activity would have waned at the expense of high HPA axis activity (i.e., the slower stress response system) [16].

Evidence for the hypothesis that the activation of the fight/flight response (i.e., SAM activity) following the introduction of a social stressor would lead to the production of a qualitatively different body odor was derived from two sources: the sender and the receiver. Three measures were used to assess whether target states were effectively induced in senders. Heart rate (HR) is a well-established non-invasive (compared to a blood test) and non-controversial measure (compared to α-amylase) of the sympathetic nervous system component of the SAM stress response [43]. The amount of armpit sweat could serve as a second indicator of SAM activity, because the adrenal medulla releases adrenalin, which activates the apocrine sweat glands in the armpit region. Third, salivary cortisol is a reliable indicator of HPA axis activity [17]. Compared to baseline, the fast stress condition was expected to be characterized by high HR and increased axillary sweat production, whereas high cortisol levels were expected to be encountered in the slow stress condition.

What is essential is that receivers were expected to display signs of a simulacrum of the state of the sender only when they were exposed to sweat obtained from participants in the fast stress condition. Since the effects induced by odors are usually hard to verbalize [44], only implicit measures of affect and behavior were included. Specifically, a simulacrum of fear was determined by (i) measuring the emergence of a fearful facial expression (i.e., increased *medial frontalis* and *corrugator supercilii* activity; cf. [3, 11, 12]) and explored further by (ii) assessing whether participants displayed increased speed and/or accuracy with regard to the classification of fearful facial expressions, compared to happy, neutral, and disgusted expressions. Since previous research reported facilitated recognition of fearful facial expressions when these expressions (i.e., visual information) were paired together with fearful voices [45], we expected that exposure to fast stress odor would result in enhanced processing of fearful facial expressions, compared to (other) negative affective expressions (disgust), positive affective expressions (happiness), and neutral expressions.

## Materials and Methods

### Ethics statement

Utrecht University Institutional Review Board approval was obtained for all experiments.

### Part 1

**Participants and design**. Eight males (“senders”; *M*_age_ = 22.50, *SD*_age_ = 3.25) provided written informed consent prior to participating. In line with previous research (see e.g. [2, 3]), we recruited only males because they have larger and more active apocrine sweat glands responsible for chemosignal production. All participants reported to be heterosexual, healthy, and non-smokers. They refrained from medication and were not diagnosed with a psychological disorder. Each participant completed three within-subjects conditions in the following order: baseline, fast stress, and slow stress.

**Materials and measures**. The Dutch version [46] of the Eysenck Personality Questionnaire-Revised Short Scale (EPQ-RSS; [47]) was administered to measure psychoticism, neuroticism, extraversion, and social desirability. The EPQ-RSS consists of 48 yes/no items. Senders’ scores on the subscales (12 items each) fell in the typical range (neuroticism: *M* = 3.75, *SD* = 2.82; psychoticism: *M* = 3.62, *SD* = 2.00; extraversion: *M* = 7.88, *SD* = 2.95; social desirability: *M* = 3.50, *SD* = 2.73).

Sweat was sampled from each axilla (armpit) on a 10 × 10 cm sterile absorbent compress (Cutisorb, BSN medical GmbH & Co KG, Hamburg, Germany) and weighed on a TP 500 pocket scale with. 01 gram precision. Sweat was sampled during 10 minutes (baseline, fast stress, slow stress). A 10 minute sweat sampling interval is likely to be sufficient as axillary stress sweat production could be as high as 32 mg/min (unpublished data, as cited in [42]) and previous research showed that receivers displayed a simulacrum of fear after exposure to “fear sweat” that weighed ~200–300 mg [11].

Heart rate was recorded with a photoplethysmograph (PPG) transducer (TSD200C, BIOPAC Systems, Inc., CA, USA) attached to the right ear lobe. Operating with a pulse plethysmogram amplifier (PPG100C), the TSD200C consists of a matched infrared emitter (wavelength: 860 nm ± 60 nm) and photo diode detector, which transmits changes in infrared reflectance resulting from varying blood flow.

Salivary cortisol was sampled while participants gently chewed on a cotton swab that was contained in a transparent plastic test tube (Salivette, Sarstedt, Newton, North Carolina). Cortisol measurement would not be affected by salivary flow rate [48].

**Procedure**. Donors followed a strict regimen to avoid sweat contamination starting two days before the sweat donation session. Alcohol use, sexual activity, odorous food consumption (e.g., garlic, onions, and asparagus), and excessive exercise were prohibited. Donors were provided with scent-free hygiene products to use in the pre-donation period. They filled in a diet diary to monitor food intake. On the donation day, donors wore a pre-washed t-shirt stored in a zip-locked plastic bag to prevent odor contamination from their clothes. From one hour before the experiment, participants were not allowed to eat food with high sugar or acidity, or take in high doses of caffeine, as this could compromise the cortisol level assay.

**Fig 1:**
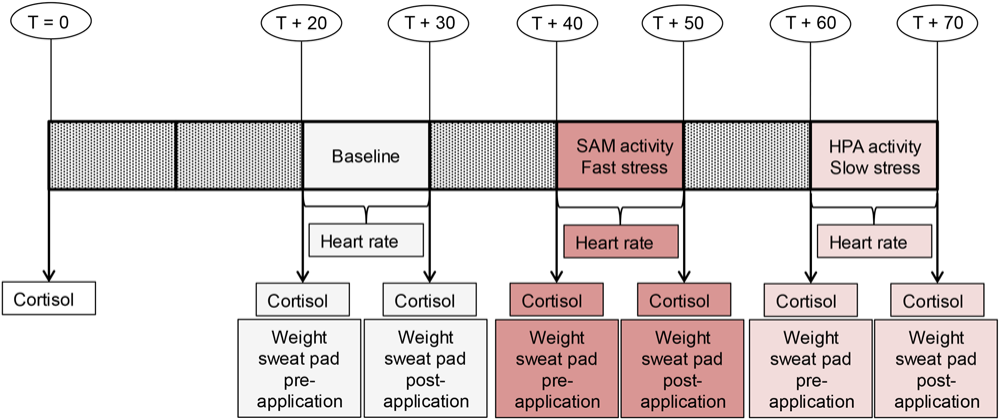
Study design Experiment Part 1. The timeline displays sweat sampling conditions: baseline, fast stress (i.e., SAM activity), slow stress (i.e., HPA axis activity), and measurements: heart rate, cortisol, sweat production. *T* = time. *T*+20 = 20 minutes passed since the start of the experiment.

The actual experiment took 70 minutes and was carried out in the afternoon (13:00–17:00), as cortisol levels would show less variation during these hours [16]. Sweat was sampled during 10 minutes on three occasions (Fig. 1): baseline, fast stress (SAM activity), and slow stress (HPA activity). The first sweat sampling session was preceded by a 20 minute wildlife documentary (BBC’s “Yellowstone, Autumn”) that was used to induce a pleasant-neutral *baseline* feeling state [49] (cf. [11, 12]). The documentary started as soon as participants had gently chewed on a Salivette for one minute. The documentary—divided into six parts—was shown during the non-sweat sampling intervals (Fig. 1) and both during the baseline condition and slow stress condition. During the fast stress condition, participants performed the anticipatory stage of the (adapted) Trier Social Stress Task [39]; they received a pre-recorded verbal instruction to prepare for an application as research assistant in front of a panel of scientists.

Prior to each sweat sampling session, donors rinsed and dried their armpits with water and paper towels. The experimenter wore vinyl gloves to avoid bacterial contamination and used hypo-allergenic tape to attach a pre-weighed pad under each armpit. Donors put on a new t-shirt and sweater before entering the cubicle (23°C) in which the experiment was run. The heart rate sensor was applied to the ear lobe. Once the experimental condition was finished, the participant called the experimenter, who removed the ear lobe sensor and sweat pads. Salivettes were frozen at -22°C. All sweat pads were weighed and stored separately in vials at -22°C. The hedonic properties and intensity of sweat would not be affected by stimulus freezing [50]. When the third sweat sampling condition was finished, donors were debriefed and they received €30 for their participation.

**Statistical analysis**. According to a Shapiro-Wilk test, cortisol, heart rate, and sweat pad weight data were normally distributed. Analysis of Salivettes was performed by a specialized relation lab of U-diagnostics (Utrecht, the Netherlands), who provided us with the cortisol levels (nmol/l). There were no missing cortisol and sweat pad weight data. Heart rate data contained substantial artifacts in a number of cases. The start and end points of these artifacts were documented and artifact deletion was applied before calculating mean heart rate (beats per minute, bpm). In two cases, heart rate data was incorrectly recorded, and stochastic regression imputation including a random error term was applied. The material of eight senders would be sufficient to present to 32 receivers in Experiment Part 2. Nevertheless, 10 participants were tested in total, because two donors did not adhere to the protocol/instructions. Their material was not used and their data were not analyzed.

### Part 2

**Participants and design**. Informed consent was obtained from 32 female participants (“receivers”); 1 participant was excluded from data analysis as this person had participated in a similar study before (*N* = 31: *M*_age_ = 21.00, *SD*_age_ = 2.02). Only females were recruited as chemosignal recipients as they generally have a better sense of smell and greater sensitivity to emotional signals (see also [2, 3]). Moreover, gender differences in chemosignal reception exist with only female receivers showing the behavioral [11] and neural [30] consequences of fear after fear sweat exposure. Participants had passed the pre-experimental screening that excluded left-handers, smokers, and individuals who had a psychological disorder, respiratory disease, illness, cold or allergy. All participants were assessed by means of a standardized psychophysical test of olfactory function (Sniffin’ Sticks, Burghart Instruments, Wedel, Germany); 29 participants had a normal sense of smell [phenethyl alcohol (PEA) threshold: *M* = 10.35 (binary dilution steps—corresponding to 6.11x10^–2^% liquid concentration), *SD* = 3.04, range: 3.25 (i.e., 8.41x10^–1^%)–15.25 (i.e., 2.05x10^–4^%)] [51] and the scores of 3 participants (≤ 5 binary dilution steps) would label them “hyposmic” [52]. Only non-smellers would be excluded and none were encountered. Participants enrolled in a counterbalanced 3 × 4 × 5 within-subjects design with odor (3 levels: baseline, fast stress, slow stress), facial expression (4 levels: fear, disgust, happiness, neutral) and the degradation level of the presented facial expression, “noise level” (5 levels: 20, 40, 60, 80, 100%), as within-subjects factors.

**Measures and materials**. Sweat pads that were obtained in the donor phase had to be prepared before presentation to receivers. The first step consisted of cutting each sweat pad (10 × 10 cm) into eight parts (12.5 cm²) with sterilized scissors. To reduce effects of inter-individual variability in sweat production, each vial that would eventually be presented to receivers contained pad parts (four in total) that came from a different donor and stemmed from either the left (two parts) or right (two parts) armpit in a pre-determined randomized order. Each participant was exposed to the same combination of pad parts across odor conditions. Odor presentation was double-blind, because each vial was marked by a code representing the odor condition by a researcher that was not involved in running the experiment.

A handedness scale was included to corroborate the right-handedness of the sample and to control for possible handedness-related differences in facial muscle activity. On a 10-item questionnaire [53] (Cronbach’s α = .98), participants indicated which hand(s) they use to perform a range of activities. All participants were right-handed (*M* = 9.39, *SD* = 1.05).

Facial electromyographic (EMG) activity was recorded bipolarly with sintered Ag/AgCl electrodes that were applied to the left side of the face—the side most strongly involved in spontaneous affective reactions in right-handed participants [54]. Following general guidelines [55], electrodes filled with hypo-allergenic conductive gel (Lectron II, Newark, NJ) were applied to the muscle that lifts the eyebrow, *medial frontalis*, and to the muscle that furrows the brow, *corrugator supercilii*. The reference electrode was placed on the middle of the forehead. EMG signals were recorded with Mindware Software (Version 2.5) and filtered online with a. 5 Hz low cutoff filter and 200 Hz high cutoff filter. The EMG signal was rectified and smoothed with a 20 Hz low pass filter with a time constant of 100 ms.

In the Noisy Facial Expression Classification Task (NFECT), participants had to classify four types of facial expressions. The expressions were negatively valenced (fear, disgust), positively valenced (happiness), and neutral. All photos stemmed from the Radboud Faces Database (RFD) [56] (codes: m23, v02, m33, v12, m25, v22, m71, v27). The facial expressions that were used in the experiment were first converted to grey-scale in Adobe Photoshop (CS6, Adobe systems Inc., San Jose, CA), after which a Gaussian noise filter (20%, 40%, 60%, 80%, 100%) was applied to only the face (i.e., not to the hair, clothes, and background). A 100% noise filter did not imply that the facial expression was invisible. However, quick stimulus presentation (50 ms) made it relatively difficult to classify the “noisier” expressions. Noise level was manipulated for exploratory purposes, namely to examine whether body odors related to fear would enhance the accuracy/speed of detection of facial cues even when these cues were difficult to detect.

This task was used to check whether static facial expressions would lead to facial mimicry. Participants viewed 24 full-colored pictures from the Radboud Faces Database (RFD) [56] (codes: m23, v02, m33, v12, m25, v22, m71, v27). Of the 24 stimuli, 12 pictures displayed a (fe)male actor; 6 faces contained a fearful, disgusted, happy, or neutral expression.

Because the emergence of a simulacrum may depend on the level of empathy of a receiver, a translated digital version of the empathy quotient (EQ) questionnaire [57] was administered. On this 60-item questionnaire (including 20 filler items), participants had to indicate on a 4-point Likert scale to what extent they agreed with statements related to empathy (0 = “strongly disagree”, 1 = “slightly disagree”, 2 = “slightly agree”, 3 = “strongly agree”). With a mean score of 47.42 (*SD* = 7.61), the current sample fell within the normal score range (<1 *SD* above the *M*; [57]).

Because the EQ may be susceptible to reporting bias, a computerized adaptation of the revised “Reading the Mind in the Eyes Task” (RMET) [58] was used to objectively assess participants’ ability to infer another person’s emotional state. The revised RMET consists of 36 photographs depicting the eye region of different (fe)male actors. Participants were asked which one of four descriptions of the photograph best described the state of the person. The current sample had an RMET score that fell in the normal range (*M* = 27.68, *SD* = 2.97) [58].

In a pre-determined counterbalanced order, participants evaluated the odors they were exposed to (baseline, fast stress, slow stress) on pleasantness and intensity (7-point Likert scale; 1 = “very unpleasant/weak”; 4 = “neither unpleasant/weak, nor pleasant/strong”; 7 = “very pleasant/strong”).

To assess participants’ ability to discriminate the presented odors, the 2-Alternative Forced-Choice Reminder (2-AFCR) task was conducted [59]. On four trials, participants indicated which of two odor stimuli (presented second or third) corresponded to the reminder (R) odor stimulus (presented first). Comparisons were made between odors obtained from the conditions: fast stress (R) and slow stress (trial 1, 2), baseline (R) and slow stress (trial 3), and baseline (R) and fast stress (trial 4). Comparison stimuli were presented in a pre-determined counterbalanced order.

Smell threshold was assessed with Sniffin’ Sticks (Burghart Instruments, Wedel, Germany), using a triple-forced choice staircase method [51]. While blindfolded, participants were presented with three markers in a row and asked to identify the single marker that contained the target smell (phenethyl alcohol). Each marker was randomly presented (2 s) about 2 cm below the nostrils of the participant. The odor concentration of the target marker was increased each time (1.22x10^–4^%-4%, with 1:2 binary dilution steps) until participants made two consecutively correct identifications, after which they were presented with a lower concentration (first reversal). If participants erred, they were again presented with a higher concentration (second reversal). The smell threshold was calculated by taking the mean of the final four (out of seven) reversal points.

Funneled post-experimental debriefing [60] revealed that 4 participants identified the odor stimulus as sweat. When probed for suspicion regarding the purpose of the study, no participant correctly guessed the hypothesis.

**Procedure**. After receiving information about the experiment, participants made an informed decision whether they wanted to participate. If yes, they filled in a screening and handedness questionnaire. Appointments were made with participants that met the inclusion criteria.

All participants provided written informed consent in the lab. A female experimenter carried out the experiment, because in body odor experiments the presence of a male experimenter was shown to increase mood in female participants [61]. Odor stimuli were defrosted 30 minutes prior to use and each participant received a new vial.

Participants received the instruction that physiological measures would be applied to their face, after which they had to perform computer tasks and a series of other tests. Participants were seated in a cubicle. The skin on the middle and left side of their forehead was cleaned with abrasive lotion (Lemon Prep, Mavidon, Lake Worth, FL) and alcohol to reduce the impedance of the EMG signal. The application of alcohol additionally served to wipe out the potentially confounding influence of Lemon Prep scent. EMG electrodes were applied next. The impedance of EMG electrodes was measured; in rare cases that the impedance exceeded 30 kΩ, an online check of the EMG signal was performed by the experimenter to determine whether the signal was reliably discernible from noise. To this end, participants had to lift (*medial frontalis*) and knit (*corrugator supercilii*) their brows—no references to emotions were made. Electrode replacement turned out to be unnecessary.

Participants sat on an adjustable chair with their head placed in a chin rest. The chin rest both stabilized the head of the participant and supported the vial that was placed about 2 cm below the nose of the participant. Before the odor-containing vial was opened, participants completed four practice trials of the NFECT. The facial expressions included fear (actor: v02, 80% noise), happiness (m23, 40%), and neutral (v02, 40%; m23, 80%) [56]. Picture presentation was controlled by Presentation software (Version 16.4) installed on a computer (19-in. screen, 1280 × 1024 screen resolution). Participants were told to classify as soon and accurately as possible the facial expression displayed on the screen as “emotion” or “no emotion” by pressing a designated key. These keys were counterbalanced across participants. The facial expression appeared on the screen for 50 ms and was preceded by a fixation cross (500 ms) and followed by a response window (maximum duration: 2 s). The inter-stimulus interval was 1.5 s.

When the practice trials were finished, participants were exposed to each of three odor stimuli (baseline, fast stress, slow stress) in a pre-determined counterbalanced order. Participants wore a nose clip to prevent preliminary sniffs. The nose clip was removed directly after the opening of the vial. The video capture embedded in EMG analysis software would reveal the moment of odor exposure (100 ms accuracy). As soon as the vial was opened, participants saw a black fixation cross that was presented on a grey background in the middle of the screen for 5 seconds. Then, they performed 40 unique trials of the NFECT. Of these 40 trials, 10 contained either a fearful, disgusted, happy, or neutral expression. Half of the trials displayed a (fe)male actor and the expression of each actor had a different level of degradation (i.e., “noise level”: 20, 40, 60, 80, 100%). When the first block of 40 trials was finished, participants took a short break. After this break, the next odor was presented and the cycle was repeated.

After three blocks, participant fulfilled 24 trials of the full facial expression classification task. The trial sequence was similar to that of the NFECT. After this task, participants performed the EQ and RMET. When participants finished the empathy questionnaires, the experimenter removed the electrodes.

In a separate room, participants were asked to rate the pleasantness and intensity of the baseline, fast stress, and slow stress odor and they had to discriminate between these odors, before they performed a smell threshold test. Finally, they completed the funneled debriefing procedure, were debriefed, thanked, and paid €12.

**Statistical analysis**. Sample size was determined by a priori power analysis (G*Power 3.1) [62] for analysis of variance, *f* = .25, power = .80, α = .05. Effect size *f* was converted [63] from the lowest effect size (η² = .06) obtained in similar research measuring the impact of sweat obtained from fearful individuals on *medial frontalis* and *corrugator supercilii* activity [11, 12]. The manner of handling the data was determined as follows. For all EMG and reaction time variables, outliers were identified with the most robust scale measure in the presence of outliers, by means of values that surpassed 2 median absolute deviation (MAD) units [64]. When outliers were revealed for a particular variable, these values were altered to be one unit above the next extreme score on that variable according to the method described in [65]. Missing values due to measurement error were handled by means of stochastic regression imputation (i.e., deterministic regression imputation with an added random error component). Removing three hyposmic individuals from the sample did not affect the outcome of the main analyses. The analyses were based on 31 participants.

## Results

### Part 1 (“senders”)

Sweat was collected from *senders* to serve as odor stimuli presented to *receivers* in Part 2. The small yet sufficient sender sample (*N* = 8) means that the results reported for this sample should be interpreted with caution. Compared to both the baseline and fast stress condition, higher heart rate and sweat production was expected in the fast stress condition (i.e., SAM activity), whereas the slow stress condition was expected to be characterized by high levels of cortisol (i.e., HPA activity).

**Fig 2:**
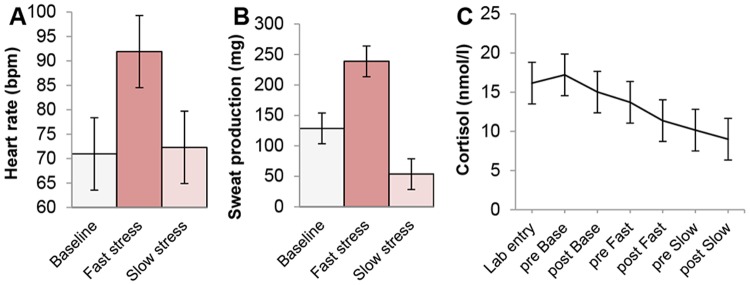
Physiological outcomes senders. Physiological measurements obtained from senders during three conditions: pleasant-neutral baseline, preparation for a speech (i.e., fast stress, SAM axis activity), and recovery from speech preparation (i.e., slow stress, HPA axis activity). (A) Mean heart rate (bpm) per condition. (B) Mean sweat production (mg) per condition. (C) Mean salivary cortisol (nmol/l) over time, including a measure following lab entry and measurements before and after each experimental condition (Base = Baseline; Fast = Fast stress; Slow = Slow stress). Error bars ± 68% within-subjects confidence interval (CI) of the main effect (for the formula leading up to the calculation of the CI, see [66]; for the choice of a 68% CI, see [67]).

A repeated measures ANOVA on heart rate with Greenhouse-Geisser correction of degrees of freedom (ε = .54) revealed a marginally significant main effect of condition (3 levels: baseline, fast stress, slow stress), *F*(2,14) = 5.21, *p* = .052, η_*p*_^2^ = .43. In line with what was expected, planned paired t-tests showed that heart rate was highest in the fast stress condition (*M =* 91.91 bpm, *SD* = 25.75 bpm) (Fig. 2A). Compared to the fast stress condition, heart rate was significantly lower in the baseline condition (*M* = 70.96, *SD* = 11.34), *t*(7) = 2.37, *p* = .050, *r*² = .45. However, a similar difference between the fast stress and slow stress condition (*M* = 72.30, *SD* = 9.68) was only marginally significant, *t*(7) = 2.25, *p* = .059. Finally, heart rate did not differ significantly between the baseline and slow stress condition, *t*(7) = .65, *p* = .54.

A rather similar pattern of results emerged for sweat production (Fig. 2B). A repeated measures ANOVA on sweat production indicated a significant main effect of condition (3 levels: baseline, fast stress, slow stress), *F*(2,14) = 14.55, *p* <. 001, η_*p*_^2^ = .68. Participants produced significantly more sweat in fast stress condition (*M* = 226.3 mg, *SD* = 142.62 mg) compared to both the baseline condition, *t*(7) = 2.60, *p* = .035, *r*² = .49 (*M* = 126.30, *SD* = 81.76), and slow stress condition, *t*(7) = 5.29, *p* = .001, *r*² = .80 (*M* = 56.20, *SD* = 43.40). Furthermore, participants perspired more in the baseline condition than in the slow stress condition, *t*(7) = 3.17, *p* = .016, *r*² = .59. Notably, heart rate did *not* differ between the baseline and slow stress condition; these apparently contradicting findings may be explained by excitement experienced by participants during the first time of sweat pad application. This excitement was not measured, as the heart rate electrode was applied after sweat pad application.

Next, salivary cortisol levels were analyzed. A repeated measures ANOVA with time (7 levels: lab entry, before baseline, after baseline, before fast stress, after fast stress, before slow stress, after slow stress) as single factor revealed a significant effect of time, *F*(6,42) = 6.24, *p* = .023, η_*p*_^2^ = .47 (ε = .24). Contrary to the expectation, participants’ cortisol levels were highest around the beginning of the experimental procedure and values decreased over time (Fig. 2C). Planned paired t-tests confirmed that compared to time point 5 to 7, higher cortisol levels were observed on time point 2 (before baseline), 3 (after baseline), and 4 (before fast stress), *p*s ≤. 30. All other comparisons (i.e., barring the comparison between time point 2 and 4, *p* = .025) were not significant, *p*s >. 05.

In sum, higher cortisol levels during the beginning (vs. end) of the experiment suggested that preparing for a speech triggered less HPA activity than being unfamiliar with the experimental procedure. Nevertheless, HPA activity was not related to axillary sweat production. Alternatively, high heart rate and sweat production—signs of sympathetic and adrenal medullary activity, respectively—were observed during speech preparation (fast stress). Arguably, SAM system activity caused the apocrine sweat glands to produce a distinctive olfactory signature of fear. Next, we examined whether the odor sampled in the fast stress condition induced in receivers a simulacrum of the senders’ experience.

### Part 2 (“receivers”)

**Fig 3:**
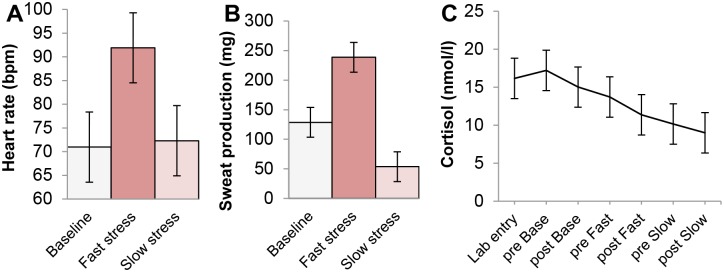
Mean facial muscle activity of receivers over time as a function of odor. (A) Mean *corrugator supercilii* activity (i.e., brow knit) following odor onset (in seconds). (B) Mean *medial frontalis* activity (i.e., brow lift) following odor onset (in seconds). Facial muscle activity displayed here was measured before the start of the facial expression classification task, to isolate the effect of odor. Error bars reflect 68% within-subjects CI of the interaction between odor and time.

Facial muscle activity indicative of a fearful expression (i.e., increased *medial frontalis* and *corrugator supercilii* activity) was first analyzed over a time window that would typically encompass two sniffs [68]. Because previous research has shown that in the context of fear odor, the second sniff (i.e., occurring ~3–5 s after odor onset) would coincide with the emergence of a fearful facial expression [3, 12], we expected a significant interaction between odor and time on both the *medial frontalis* and *corrugator supercilii* muscle (see [69], for a list of facial muscles related to emotion categories, and see [70] for a critique on whether facial EMG can be used to discriminate discrete emotions in an emotional facial mimicry setting). A 3 × 5 repeated measures ANOVA on mean *corrugator supercilii* activity with odor (3 levels: baseline, fast stress, slow stress) and time (5 levels: 0–1 s, 1–2 s, 2–3 s, 3–4 s, 4–5 s) as factors revealed a significant main effect of odor, *F*(2,60) = 3.30, *p* = .044, η_*p*_^2^ = .10, and time, *F*(4,120) = 20.06, *p* <. 001 (ε = .66). As predicted, the main effects were qualified by an interaction between odor and time, *F*(8,240) = 3.27, *p* = .013, η_*p*_^2^ = .10 (ε = .52) (Fig. 3A). Follow-up non-parametric Wilcoxon signed-rank tests indicated significantly higher *corrugator supercilii* activity in the 4^th^ and 5^th^ second after fast stress odor onset, compared to baseline, *Z* = 1.86, *p* = .063; *Z* = 2.14, *p* = .033, and slow stress, *Z* = 2.14, *p* = .033; *Z* = 2.04, *p* = .042. In addition, lower *corrugator supercilii* activity was encountered in the baseline condition compared to the slow stress, *Z* = -2.37, *p* = .018, and fast stress condition, *Z* = -2.12, *p* = .034. Other comparisons did not yield significant differences, *p* >. 05.

Another 3 × 5 repeated measures ANOVA on *medial frontalis* activity indicated a marginally significant effect of odor, *F*(2,60) = 2.71, *p* = .075, η_*p*_^2^ = .08, and a significant effect of time, *F*(4,120) = 7.24, *p* = .002 (ε = .50). More importantly, these effects were qualified by an interaction between odor and time, *F*(8,240) = 2.46, *p* = .040, η_*p*_^2^ = .08 (ε = .58) (Fig. 3B). Follow-up Wilcoxon signed-ranks tests indicated significantly higher *medial frontalis* activity on the 2^nd^ second following fast stress odor onset compared to baseline, *Z* = 2.55, *p* = .011 (comparison between slow stress and baseline: *Z* = 1.88, *p* = .06). The other comparisons were not significant, *p* >. 05.

**Fig 4:**
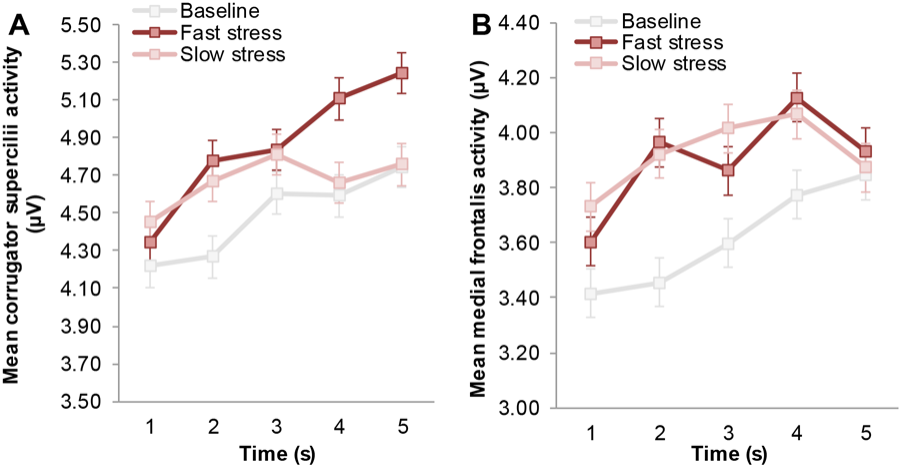
Mean facial muscle co-activation of receivers over time as a function of odor. Facial muscle activity displayed here was measured before the start of the facial expression classification task, to isolate the effect of odor. Above each bar, the time after odor onset (in seconds) is depicted (see Y-axis). The more each bar is located toward the upper-right end point (vs. bottom-left starting point) of the dashed diagonal, the more the *medial frontalis* and *corrugator supercilii* muscles co-activated (μV), resembling a fearful facial expression [cf. 11, 12].

Taken together, the present findings indicate that highest co-activation of the *medial frontalis* and *corrugator supercilii* muscle indicative of fear occurred after exposure to fast stress odor (Fig. 4). Aside from the five seconds directly following odor onset, facial EMG was measured while participants performed the emotional facial expression classification task (NFECT). Receivers were expected to show increased *corrugator supercilii* and *medial frontalis* activity when fearful facial expressions were presented in the context of fast stress odor.

**Fig 5:**
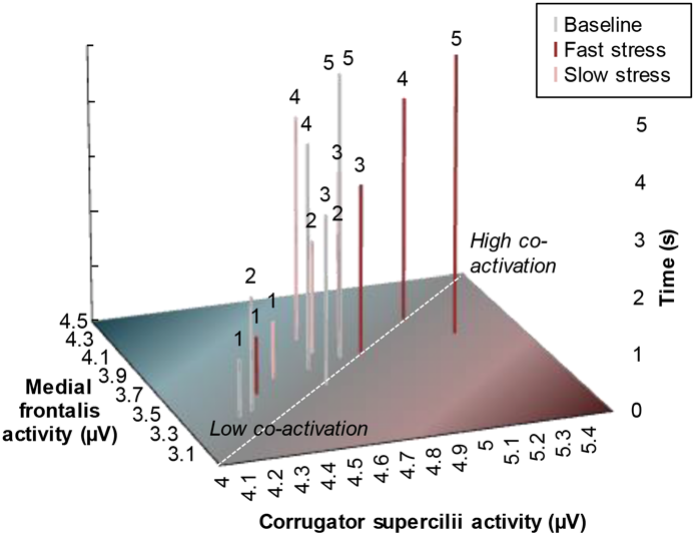
Mean facial muscle activity of receivers per odor condition during classification of presented (emotional) facial expressions. Odor condition: Baseline, fast stress, slow stress. Facial expressions that had to be classified: Neutral, happy, fear, disgust. For clarification purposes, the display of mean facial muscle activity on the emotional facial expression classification task was collapsed over the variable noise level (20%, 40%, 60%, 80%, 100%). (A) Mean *corrugator supercilii* activity, averaged over 1 second following the onset of the presented expression. (B) Mean *medial frontalis* activity, averaged over 1 second following the onset of the presented expression. Error bars reflect 68% within-subjects CI of the main effect of odor.

First, a 3 × 4 × 5 repeated measures ANOVA on *corrugator supercilii* activity with factors odor (3 levels: baseline, fast stress, slow stress), facial expression (4 levels: fear, disgust, happy, neutral), and noise level (5 levels: 20, 40, 60, 80, 100%) revealed a main effect of odor, *F*(2,60) = 5.82, *p* = .005, η_*p*_^2^ = .16. Planned post hoc tests revealed *corrugator supercilii* activity that mirrored the pattern of the first five seconds after odor onset, with higher activity in the fast stress condition (*M* = 5.93 μV, *SE* = .56 μV) compared to the baseline, *p* = .008 (*M* = 5.42, *SE* = .47), and slow stress condition, *p* = .013 (*M* = 5.38, *SE* = .45) (Fig. 5A; cf. Fig. 3A). However, there was neither an interaction between odor and facial expression, *F*(6,180) = 2.21, *p* = .068 (ε = .70), which was contrary to our expectation, nor was there a significant three-way interaction between odor, facial expression, and noise level, *F*(24,720) = 2.01, *p* = .060 (ε = .27). In addition, noise level did not significantly impact mean *corrugator supercilii* activity, *F* < 1. However, the repeated measures analysis did reveal a main effect of facial expression, *F*(3,90), *p* = .002, η_*p*_^2^ = .17 (ε = .73). Post hoc tests indicated that highest levels of *corrugator supercilii* activity were measured while participants classified neutral faces (*M* = 5.66, *SE* = .50) compared to happy, *p* = .004 (*M* = 5.52, *SE* = .48), fearful, *p* = .007 (*M* = 5.53, *SE* = .47), and disgusted faces, *p* = .033 (*M* = 5.59, *SE* = .49). Participants presented with happy facial expressions additionally showed lower *corrugator supercilii* activity compared to disgust, *p* = .042.

Although facial muscle activity patterns reported for the *corrugator supercilii* muscle were similar to *medial frontalis* muscle activity patterns, our hypotheses were not confirmed. Another 3 × 4 × 5 repeated measures ANOVA on *medial frontalis* activity indicated a non-significant effect of odor, *F*(2,60) = 2.36, *p* = .111 (Fig. 5B). Contrary to what was expected, the interaction between odor and facial expression was not significant, *F* < 1. Furthermore, the three-way interaction between odor, facial expression, and noise, *F*(24,720) = 2.10, *p* = .062 (ε = .23) did not exceed the threshold of significance. However, the interaction between facial expression and noise level was significant, *F*(12,360) = 3.04, *p* = .009, η_*p*_^2^ = .09 (ε = .46). An examination of the remaining main effects revealed that there was no significant effect of noise level on *medial frontalis* activity, *F* < 1. Nevertheless, there was a significant main effect of facial expression, *F*(3,90) = 4.84, *p* = .004, η_*p*_^2^ = .14. Participants displayed higher *medial frontalis* activity following the presentation of a fearful facial expression (*M* = 3.72, *SE* = .36), compared to a neutral, *p* = .012 (*M* = 3.67, *SE* = .35), disgusted, *p* = .003 (*M* = 3.66, *SE* = .35), and happy expression, *p* = .067 (non-significant trend) (*M* = 3.69, *SE* = .35).

In sum, the pattern of facial muscle activity that was established in the first five seconds after odor onset was maintained during the task, a finding that replicates previous research [3, 11]. The next analysis was performed to examine whether receivers exposed to fast stress odor would show signs of increased speed and/or accuracy with regard to the classification of fearful facial expressions.

**Fig 6:**
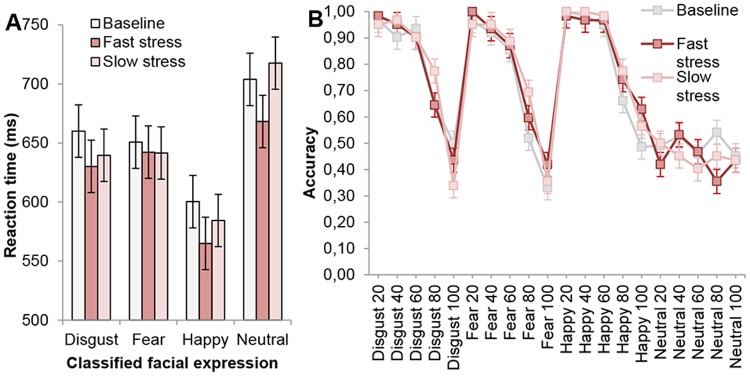
Mean speed and accuracy of receivers to classify (degraded) facial expressions per odor condition. (A) Mean reaction time (ms) of facial expression classification (disgust, fear, happy, neutral) per odor condition (baseline, fast stress, slow stress), collapsed over noise levels (for clarification purposes). (B) Mean accuracy (proportion) of facial expression classification (disgust, fear, happy, neutral) per odor condition (baseline, fast stress, slow stress), and noise level (20–100%). Error bars reflect 68% within-subjects CI of the interaction between odor and facial expression.

A 3 × 4 × 5 repeated measures ANOVA on reaction time (RT) with factors odor (3 levels: baseline, fast stress, slow stress), facial expression (4 levels: fear, disgust, happy, neutral), and noise level (5 levels: 20, 40, 60, 80, 100%) yielded a significant main effect of odor, *F*(2,60) = 3.24, *p* = .046, η_*p*_^2^ = .10, facial expression, *F*(3,90) = 35.58, *p* <. 001, η_*p*_^2^ = .54, and noise level, *F*(4,120) = 80.90, *p* <. 001, η_*p*_^2^ = .73 (ε = .53). A planned post hoc test following up on the main effect of odor indicated that participants were significantly faster in classifying *all* facial expressions in the fast stress condition (*M* = 627.17 ms, *SE* = 15.62 ms) compared to the baseline, *p* = .019 (*M* = 656.69, *SE* = 17.78) and slow stress condition, *p* = .068 (non-significant trend) (*M* = 646.76, *SE* = 13.22) (see Fig. 6A). RT did not significantly differ between the baseline and slow stress condition, *p* = .451. The three-way interaction between odor, facial expression, and noise level was not significant, *F*(24,720) = 1.70, *p* = .074 (ε = .45). Concerning the two-way interactions, only the interaction between facial expression and noise level was significant, *F*(12,360) = 9.19, *p* <. 001. A post hoc test following up on the main effect of facial expression indicated a typical response pattern (cf. [71]). That is, participants were significantly faster in detecting happy facial expressions (*M* = 584.93, *SE* = 12.10) compared to neutral (*M* = 697.93, *SE* = 15.59), fearful (*M* = 645.49, *SE* = 17.77) and disgusted (*M* = 645.80, *SE* = 16.40) expressions, *p*s <. 001. At the same time, participants were significantly slower in detecting neutral expressions relative to expressions that contained emotional expressions, *p*s <. 001. Finally, a post hoc test on noise level indicated that receivers showed the fastest classification responses when expressions were least degraded: 20% (*M* = 558.12, *SE* = 10.16), followed by 40% (*M* = 594.39, *SE* = 12.78), 60% (*M* = 636.17, *SE* = 12.94), 80% (*M* = 706.41, *SE* = 18.41), and 100% noise (*M* = 722.61, *SE* = 21.60); all comparisons (i.e., barring the comparison between 80–100%), *p* <. 001.

Another 3 × 4 × 5 repeated measures ANOVA on accuracy with odor (3 levels: baseline, fast stress, slow stress), facial expression (4 levels: fear, disgust, happy, neutral), and noise level (5 levels: 20, 40, 60, 80, 100%) as factors did not reveal a significant main effect of odor, *F* < 1, yet there was a main effect of facial expression, *F*(3,90) = 19.44, *p* <. 001 (ε = .42), and noise level, *F*(4,120) = 113.93, *p* <. 001 (ε = .50) (Fig. 6B). Follow-up post hoc tests on facial expression revealed that classification of happy facial expressions occurred with the highest accuracy compared to all other expressions, *p*s <. 016 (happy: *M* = .85, *SE* = .02; disgust: *M* = .79, *SE* = .02; fear: *M* = .75, *SE* = .02). In contrast, classification of neutral expressions occurred with the lowest accuracy, *p*s <. 001 (neutral: *M* = .47, *SE* = .07). Furthermore, post hoc tests on noise level indicated that participants displayed significantly lower accuracies (i.e., barring the 20–40% comparison) with each decrement in noise level, *p*s <. 009 (100%: *M* = .45, *SE* = .03; 80%: *M* = .63, *SE* = .03; 60%: *M* = .81, *SE* = .02; 40%: *M* = .84, *SE* = .02; 20%: *M* = .85, *SE* = .02). Concerning the two-way interactions, the only significant interaction was between facial expression and noise level, *F*(12,360) = 15.96, *p* <. 001 (ε = .51). Finally, the three-way interaction between odor, facial expression, and noise level was not significant, *F*(24,720) = 1.04, *p* = .41 (ε = .47).

In sum, receivers exposed to fast stress odor demonstrated a *general* increase in speed (vs. accuracy) with regard to the classification of *all* facial expressions. Hence, fast stress odor appeared to have induced sensory vigilance—replicating previous research findings [3].

To assess whether receivers mimicked the fully visible facial expressions (fear, disgust, happy, neutral) of the actors in the NFECT, another repeated measures ANOVA was conducted. Negatively valenced expressions (i.e., fear, disgust) were expected to lead to significantly higher *corrugator supercilii* activity compared to happy and neutral, whereas fearful facial expressions were expected to induce increased *medial frontalis* activity. Although a main effect of *medial frontalis* activity was encountered, *F*(3,90) = 3.16, *p* = .049 (ε = .67), the results were not in line with our hypothesis, as *medial frontalis* activity was significantly lower in the neutral condition compared to all other conditions, *p*s <. 039 (neutral: *M* = 3.46 μV, *SE* = .31 μV; fear: *M* = 3.51, *SE* = .31; happy: *M* = 3.55, *SE* = .31; disgust: *M* = 3.53, *SE* = .31); all other comparisons were not significant. Furthermore, no main effect of *corrugator supercilii* activity emerged, *F*(3,90) = 1.17, *p* = .32 (ε = .64) (neutral: *M* = 5.48 μV, *SE* = .52 μV; fear: *M* = 5.45, *SE* = .52; happy: *M* = 5.35, *SE* = .53; disgust: *M* = 5.45, *SE* = .52). The absence of evidence for facial mimicry during the classification of facial expressions might be due to the absence of a social context in which mimicry would normally occur [70].

Additional analyses were performed on participants’ ratings of the pleasantness and intensity of body odor to demonstrate that these variables did not drive facial EMG responses (Table 1). A repeated measures ANOVA on self-reported pleasantness revealed a non-significant effect of odor, *F*(2,60) = .79, *p* = .458. Another repeated measures ANOVA on intensity revealed a non-significant trend, *F*(2,60) = 2.74, *p* = .077 (Table 1). Furthermore, participants could not discriminate the baseline, fast stress, and slow stress odor, as was demonstrated by binomial analysis of odor discrimination task scores (H_0_ = 50% correct), *p*s >. 071 (% correct, Trial 1–4: 52%, 32%, 58%, 58%, respectively). Hence, concurring with what was reported in previous research (e.g. [2, 11, 12]) there is no evidence for glaring differences in the perceptual properties of the odors that may have accounted for differences in facial EMG responses; odor-evoked facial muscle responses were not relatable to verbal reports.

**Table 1:** Receivers’ ratings of sweat sampled from senders under different conditions.

	Baseline	Fast stress	Slow stress
**Intensity**	4.03 (1.60)	4.61 (1.20)	4.42 (1.63)
**Pleasantness**	3.35 (1.14)	3.13 (1.15)	3.19 (1.22)

Mean (SD) intensity and pleasantness ratings (7-point Likert scales) of sweat presented to receivers sampled from senders during different phases (baseline, fast stress, slow stress).

## Discussion

By demonstrating that receivers showed a simulacrum of the fear experience of senders, the current results confirmed the main hypothesis that the release of what has been labeled fear sweat is driven by a rapidly activated stress response in the sender that is part of the fight/flight response, namely the sympathetic-adrenal medullary (SAM) system. Experiment Part 1 showed that when participants had to prepare for a speech, heart rate was higher and sweat production was increased relative to a baseline and slow stress (i.e., later time interval) condition. Specifically, activation of the sympathetic part of the SAM system resulted in increased heart rate, and since the adrenal medulla is part of the SAM system, the subsequent release of adrenalin arguably activated the apocrine sweat glands. Sweat sampled in the fast stress condition contained a distinctive signature, as receivers exposed to this odor showed a fearful facial expression (i.e., co-activation of *corrugator supercilii* and *medial frontalis* muscle) and vigilant behavior (i.e., faster reaction times in general when classifying facial expressions). Notably, the facial expression that emerged in the five seconds after odor onset was maintained for several minutes during the task, replicating previous research [3, 12]. The combined results suggest that SAM activity results in the release of a *qualitatively* different odor stimulus by the sender, one that has a distinctive and communicable chemical signature capable of inducing in receivers a simulacrum of fear.

The current results were interpreted by combining evidence from multiple variables (i.e., facial EMG, RT) [72] as opposed to examining each variable in isolation. For instance, when *medial frontalis* activity is examined in isolation, the absence of a significant difference between the fast stress and slow stress condition could be problematic with regard to our conclusion that receivers displayed a simulacrum of fear after fast stress odor exposure. Many reasons can be provided for the absence of a difference, such as the occurrence of an orientation response [73] that would briefly emerge in case the odor exceeded a certain level of intensity (cf. Table 1). However, this explanation is less likely when facial EMG data and vigilant behavior are examined together. The advantage of this approach is that it increases the likelihood that one particular type of behavior is observed (e.g., fear) and not another (e.g., disgust, anger, or an orientation response) [72].

Although the current research provided information about the time course and physiology of “fear odor” release by senders, many epistemic gaps still need to be filled on the part of the receiver. First, a particular odor, such as fear odor, is assumed to have a distinctive signature, in the sense that it is comprised of distinctive chemical compounds, similar to what other researchers have demonstrated for gender [74], individuals [74] and disease [19]. Arguably, specific odor compounds related to fear may have become associated with particular situations in which they naturally occur (e.g., fear-inducing contexts such as academic examinations). Subsequently, a population of multimodal neurons, processing not only olfactory (i.e., *odor object* information [75]) but also auditory and visual information, may be involved in creating a representation of this event [76]. Encountering fear odor may then reactivate these previously stored representations.

Since the original state is never completely reinstated in a receiver [76], exposure to compounds related to fear induce a simulation of fear that is *partial*. A partial simulation of fear may include activation of (parts of) a neuro-motor program of fear, resulting for instance in the emergence of a fearful facial expression (e.g., [77]). Taking on a fearful facial expression could be vital in dangerous situations, as lifting the eyebrows (i.e., *medial frontalis*) would lead to increased visual field size and enhanced sensory intake [78]. The results obtained in previous research [3] and that of the current research seem to fit with this perspective, as exposure to fear odor elicited not only a facial expression of fear, but also behavior indicative of sensory vigilance. Vigilant behavior ranged from more effective eye movements and enhanced performance on an easy visual search task in previous research [3] to increased speed of classifying facial expressions in the present research.

Previous research reported facilitated recognition of fearful facial expressions when these expressions were presented together with fearful voices [45]. In a similar vein, we expected that exposure to fast stress odor (i.e., olfactory modality), would led to faster and/or more accurate detection of fearful facial expressions (i.e., visual modality). However, the results of the current research did not confirm this hypothesis. Previous research showed that exposure to sweat obtained from senders induced to be in a fearful state increased amygdala activity in (female) receivers [1, 30]. A locationist perspective invites relating the amygdala to fear behavior (e.g., [79]). Alternatively, the amygdala was suggested to play a role in fixating on and paying attention to visual information of all facial emotions [80]. The amygdala was argued to be neither a fear module, nor a negative-emotions-dedicated subsystem, but rather a relevance detector [81]. The psychological constructionist view similarly states that the amygdala is part of a network that signals the motivational salience of the stimulus [82]. Hence, similar to what has been observed in previous research [1, 30], the motivational salience of the fast stress odor may be processed, amongst other brain regions, at the level of the amygdala. What followed was increased vigilance toward (other) relevant social stimuli in the environment (e.g., facial expressions), ultimately resulting in increased classification speed of *all* facial expressions.

One can ask whether the experience of receivers, and that of senders, can be classified as the emotion category *fear*. Labeling particular behaviors and facial expressions as discretely fearful may not match with the person’s actual experience [83]. Instead, emotions were argued to be rooted in *core affect*, characterized by the components valence and arousal (e.g. [83, 84]). Emotion categories such as fear would emerge from the interplay of these basic operations in part determined by the content of the experience and the context in which the experience occurs [82]. With regard to the current research, *senders* that produced sweat in the context of preparing for a speech may have labeled their arousing negative affective experience as “fear” or “anxiety”. However, apart from the odor itself, receivers exposed to fast stress sweat had no clear-cut contextual information to make sense of their experience, which leaves open the question whether there is *emotion-specificity* in the response of receivers. Future research could examine whether exposure to odors from senders experiencing anger—another emotion category related to sympathetic-adrenal medullary (SAM) activity—leads to the release of markedly different odor compounds to which receivers respond in an emotion-specific manner. If receivers do not display emotion-specificity, then this may require rethinking the *signaling* value of chemosignals. What is then chemically transferred between a sender and receiver may not be a discrete emotion package, but rather a *cue* that elicits particular behavior based on, for instance, the receiver’s previously stored association with the odor. Notably, a *cue* could acquire *signal* properties, in case *many* individuals have stored fear odor objects. In that case, the odor emitted by one person as a function of fear may trigger in another person a partial simulation of their previously stored fear experience.

Although previous research provided neural and behavioral evidence showing that receivers displayed a *simulacrum* of the fear experience following exposure to sweat sampled from fearful senders (e.g., [1–4], remarkably little was known about the time course and physiological mechanism responsible for the release of this apparently distinctive chemical signature. The current research provided evidence suggesting that the release of that what has been labeled *fear sweat* is driven by rapid physiological changes that accompany a well-known concomitant of fear, the fight/flight response [9]. Indeed, patterns of facial muscle activity that were observed in receivers following fast stress (i.e., sampled from senders during 10 minutes) odor onset resembled patterns obtained in previous research using sampling intervals that were on average three times longer [3, 11, 12]. Hence, the apparently distinctive fear signature was created relatively rapidly as a function of the SAM system. Based on the current findings and previous research showing that sweat sampled during an interval marked by the absence of a cortisol response still induced fear-related responses in receivers [38], we presume that not HPA axis, but SAM axis activity was responsible for the chemical transfer of fear from sender to receiver.

Chemical analysis needs to determine the relation between SAM activity and the apparent emergence of distinctive sweat compounds. By narrowing down on the common physiological process (i.e., SAM activity) underlying fear/anxiety sweat production, the current research provided guidelines for future research to become more effective in inducing and measuring the *particular* state that drives the release of so-called fear/anxiety sweat. Furthermore, given the relatively rapid operation of the SAM axis, the current research has implications with regard to sampling time. Previous research demonstrated that sampling time (12 versus 24 hours) was an important determinant of the pleasantness and intensity of body odor samples [85], yet the present research adds to these findings that regardless of differences in pleasantness and intensity, changes in body odor composition may occur during relatively short time intervals. In sum, the changes implied by the present research are not only theoretical but also methodological.

Based on the steadily increasing number of contributions to the topic, research on emotional chemosignaling can be considered an emerging field. Despite the fact that pheromone communication is unlikely in humans (given the lack of a functional vomeronasal organ [86]), odors may still affect humans in a consistent manner by means of associations that emerge between an odor and the context in which the odor is typically emitted and experienced. The present research is one of a number of studies demonstrating that the sense of smell is more important than usually assumed. Although humans have difficulty naming even the most common odors [44], an estimated one trillion plus odors can be discriminated [87]. By delving into the underlying physiological mechanism of fear chemosignaling, the current research opened new lines of research that could further our understanding about how humans transfer information to each other by using the sense of smell.
