# Correction: Rapid Stress System Drives Chemical Transfer of Fear from Sender to Receiver

**DOI:** 10.1371/journal.pone.0128717

**Published:** 2015-05-21

**Authors:** The PLOS ONE Staff

The images for Figs 3, 4 and 5 are incorrect. The publisher apologizes for the error.

Please see the complete, corrected Fig 3 here.

**Fig 3:**
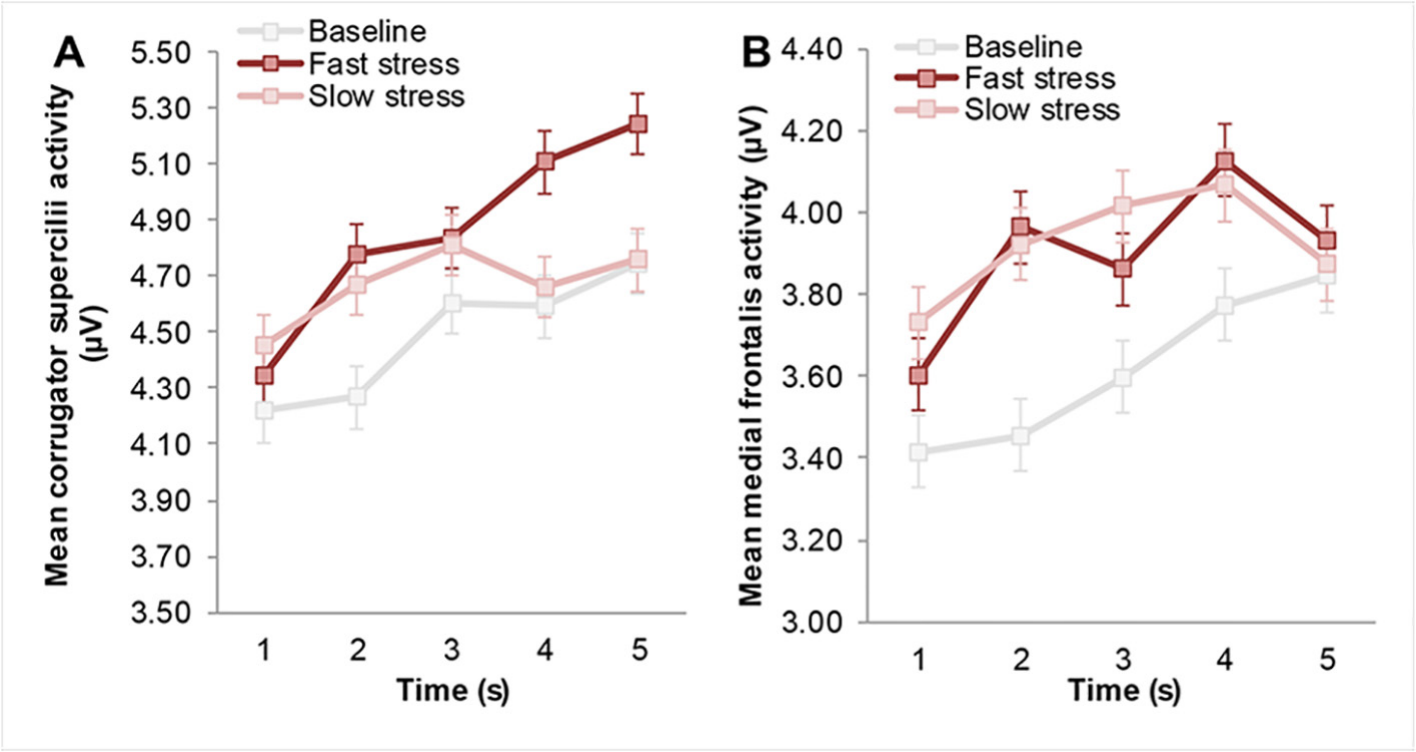
Mean facial muscle activity of receivers over time as a function of odor. (A) Mean *corrugator supercilii* activity (i.e., brow knit) following odor onset (in seconds). (B) Mean *medial frontalis* activity (i.e., brow lift) following odor onset (in seconds). Facial muscle activity displayed here was measured before the start of the facial expression classification task, to isolate the effect of odor. Error bars reflect 68% within-subjects CI of the interaction between odor and time.

Please see the complete, corrected Fig 4 here.

**Fig 4:**
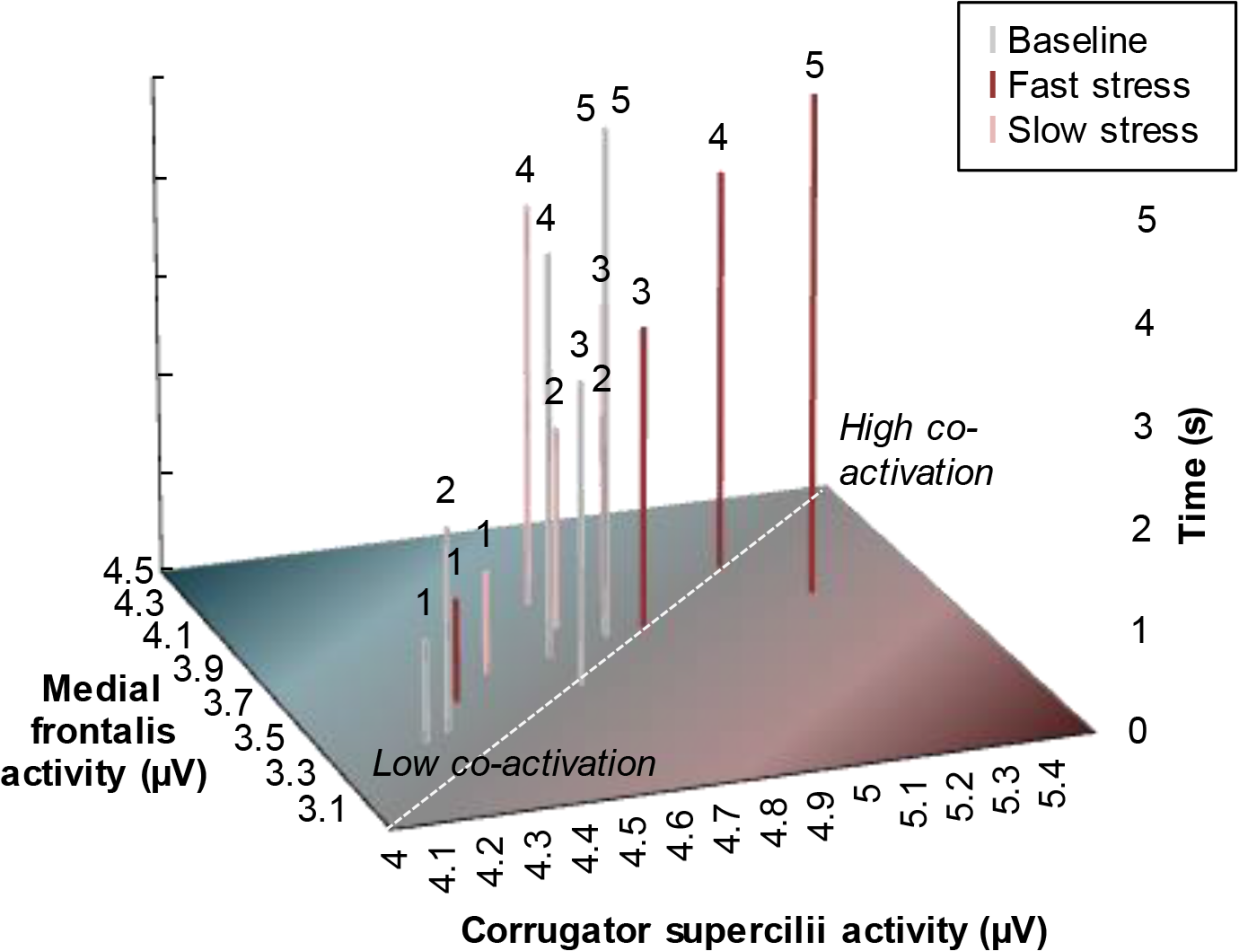
Mean facial muscle co-activation of receivers over time as a function of odor. Facial muscle activity displayed here was measured before the start of the facial expression classification task, to isolate the effect of odor. Above each bar, the time after odor onset (in seconds) is depicted (see Y-axis). The more each bar is located toward the upper-right end point (vs. bottom-left starting point) of the dashed diagonal, the more the *medial frontalis* and *corrugator supercilii* muscles co-activated (μV), resembling a fearful facial expression [cf. 11, 12].

Please see the complete, corrected Fig 5 here.

**Fig 5:**
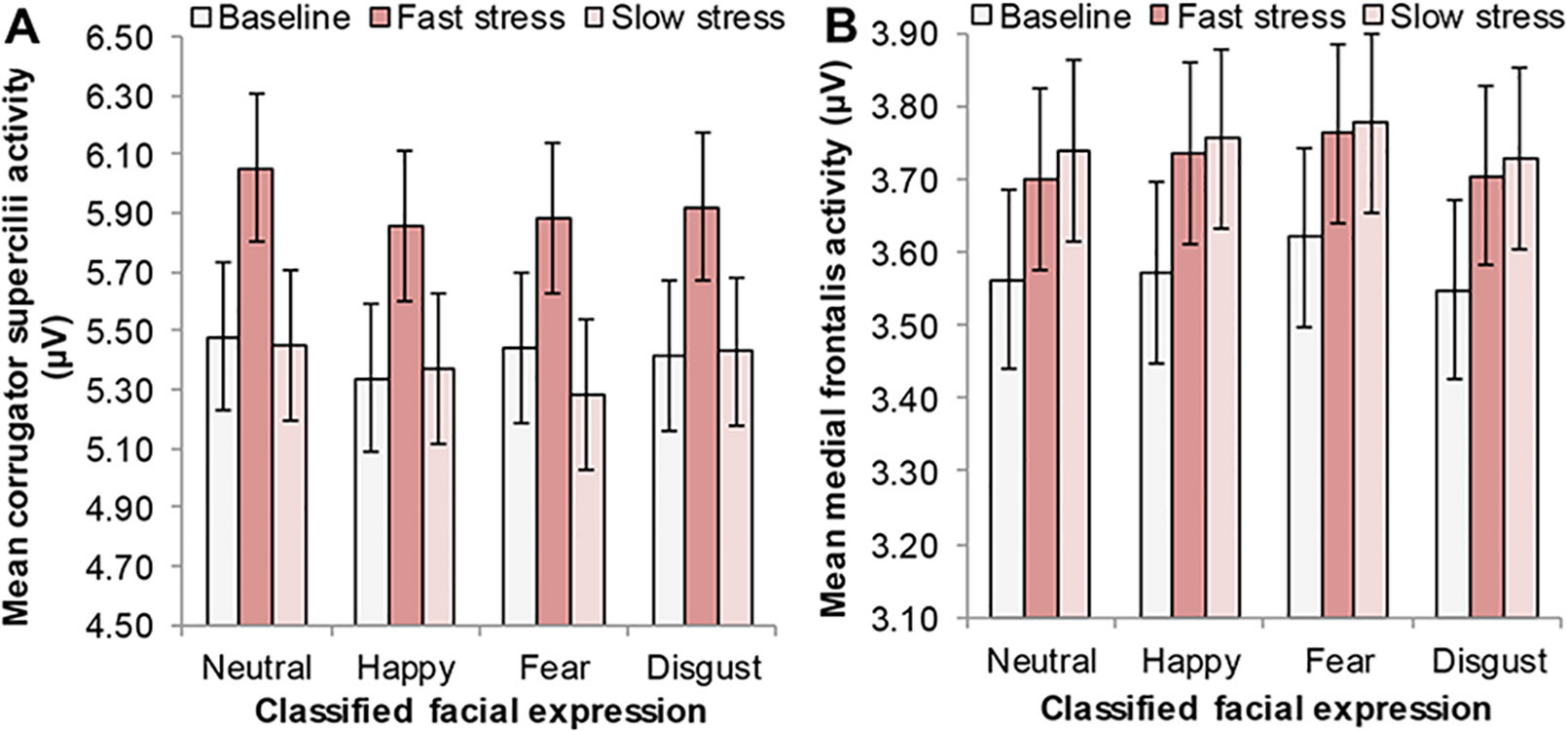
Mean facial muscle activity of receivers per odor condition during classification of presented (emotional) facial expressions. Odor condition: Baseline, fast stress, slow stress. Facial expressions that had to be classified: Neutral, happy, fear, disgust. For clarification purposes, the display of mean facial muscle activity on the emotional facial expression classification task was collapsed over the variable noise level (20%, 40%, 60%, 80%, 100%). (A) Mean *corrugator supercilii* activity, averaged over 1 second following the onset of the presented expression. (B) Mean *medial frontalis* activity, averaged over 1 second following the onset of the presented expression. Error bars reflect 68% within-subjects CI of the main effect of odor.
